# The Potential of Self-Managed Abortion to Expand Abortion Access in Humanitarian Contexts

**DOI:** 10.3389/fgwh.2021.681039

**Published:** 2021-08-13

**Authors:** Ruvani Jayaweera, Bill Powell, Caitlin Gerdts, Jessica Kakesa, Ramatou Ouedraogo, Uwezo Ramazani, Yohannes Dibaba Wado, Erin Wheeler, Tamara Fetters

**Affiliations:** ^1^Ibis Reproductive Health, Oakland, CA, United States; ^2^Ipas, Chapel Hill, NC, United States; ^3^International Rescue Committee, Kinshasa, Democratic Republic of the Congo; ^4^African Population and Health Research Center, Nairobi, Kenya; ^5^Resilience Action International, Kakuma, Kenya; ^6^International Rescue Committee, New York, NY, United States

**Keywords:** abortion, self-managed abortion, humanitarian contexts, refugees, self-care interventions, safe abortion care, humanitarian crises

## Abstract

Refugees and displaced people face uniquely challenging barriers to abortion access, including the collapse of health systems, statelessness, and a lack of prioritization of sexual and reproductive health services by humanitarian agencies. This article summarizes the evidence around abortion access in humanitarian contexts, and highlights the opportunities for interventions that could increase knowledge and support around self-managed abortion. We explore how lessons learned from other contexts can be applied to the development of effective interventions to reduce abortion-related morbidity and mortality, and may improve access to information about safe methods of abortion, including self-management, in humanitarian settings. We conclude by laying out a forward-thinking research agenda that addresses gaps in our knowledge around abortion access and experiences in humanitarian contexts.

## Introduction

The ability to control one's fertility is a fundamental human right (1, 2). Unfortunately, this right is not universally enjoyed or accessible to all people, and reproductive oppression—the control and exploitation of women, girls, and individuals through their bodies, sexuality, labor, and reproduction—persists globally. The consequences of this oppression are inequitably magnified by statelessness, disrupted communities, and health systems. The World Health Organization (WHO) estimates that almost all of the annual 25.1 million unsafe abortions globally occur in low and middle income regions; unsafe abortion is responsible for an estimated 8–13% of global maternal deaths, with low and middle-income country-specific rates frequently much higher (3). Little is known about the magnitude of unsafe abortion and its associated outcomes in humanitarian settings, although both are thought to be much worse (4). While the need for abortion services likely increases during humanitarian crises, the abortion needs and experiences of people living in humanitarian settings are often ignored. Expanding access to abortion information, support, and services is critical to ensuring the reproductive autonomy of individuals in crisis settings, yet it is rarely prioritized.

Interventions that support people who are self-managing an abortion with misoprostol alone, or in combination with mifepristone, have the potential to dramatically increase safe abortion access (5). This type of autonomous management is commonly referred to as self-care. Self-care is defined by the WHO as “the ability of individuals, families and communities to promote health, prevent disease, maintain health, and cope with illness and disability with or without the support of a healthcare provider,” and includes self-managed abortion with medications (SMA) as one of its recommended interventions (6). However, further research is needed to fully understand the scope of barriers and facilitators to increase access to self-managed abortion information and support for refugees and displaced people. This paper addresses the research gaps in our current understanding of abortion access in humanitarian contexts, explores existing barriers to safe abortion care in these settings, highlights the potential of SMA as a person-centered strategy to increase reproductive autonomy, and proposes priorities for future research in humanitarian contexts.

## Abortion in Humanitarian Contexts

There is little to no published data documenting the incidence of or experiences with abortion among individuals living in refugee camps or settlements. Given what we know about the nature of humanitarian emergencies, the need for abortion services likely increases due to the collapse of health systems, disruptions in contraceptive use and access, and increased exposure to sexual violence or transactional sex (4). As a result, displaced and conflict-affected people may be at increased risk of the consequences of lack of abortion care access, including forced childbearing, and morbidity and mortality related to unsafe abortion. An estimated 61% of maternal deaths occur in fragile states, many of which are affected by conflict and recurring natural disasters. However, accurate estimation in individual conflict-affected areas remains a challenge. Recent studies have documented a nearly 2-fold increase in post-abortion care utilization between 2012–2013 and 2015–2017 in the Democratic Republic of Congo, Somalia, and Yemen, highlighting the critical role that comprehensive safe abortion services could play (7, 8).

Despite the confluence of factors that highlight the need to prioritize abortion access, lack of research on the need for abortion services, misconceptions about the legality of abortion provision, lack of funding and donor attention, limited trained providers, and misperceptions around the technical difficulty of abortion care all serve as barriers to abortion provision from humanitarian organizations (4). Gaps in the health system, lack of commodities, lack of knowledge about the legal status of abortion and where to obtain safe services (particularly for those who are displaced across country borders), and high stigmatization of abortion are additional barriers specific to displaced people. In light of these challenges, the Inter-Agency Working Group on Reproductive Health in Crisis (IAWG) has developed a comprehensive field manual on sexual and reproductive health, which has included stand-alone chapters on safe abortion since 2010, and successfully advocated to include safe abortion in the Minimum Initial Service Package (MISP) for Sexual and Reproductive Health in 2018 (9, 10). While the MISP includes safe abortion as a priority activity, safe abortion services are routinely excluded from reproductive health service provision in humanitarian settings, and research has shown that abortion care is almost non-existent in humanitarian programming or proposals (11, 12). Citing their professional and moral responsibility to reducing maternal mortality, some humanitarian agencies, such as Médecins Sans Frontières, have explicitly stated their commitment to providing comprehensive abortion care in spite of global policy and legal barriers (13), and the International Rescue Committee has prioritized SMA in humanitarian settings as an organizational research and innovation priority. While there is some momentum in the provision of abortion care in these settings, progress has been slow, even as more nations expand the legal indications for abortion. Given these unique challenges, targeted interventions designed to increase abortion access are needed.

Research conducted among Congolese refugees in Uganda suggest many were unable to navigate the legal restrictions on abortion in that country and were instead engaging in unsafe abortion practices, such as ingesting detergents or pain medications or inserting crushed bottles and sticks into the uterus; legal restrictions on induced abortion also posed a barrier to the provision of post-abortion care (14). Additionally, recent studies on the experience of training and implementing safe abortion services in Bangladesh to Rohingya refugees highlights the immense need for abortion services: less than two years after the influx of refugees, almost 8,000 people had received abortion-related care, over 75% of which were legal induced abortions (15, 16). This case study from Bangladesh translates evidence-based findings into common practice and documents the first time legal induced abortion care, in the form of menstrual regulation in Bangladesh, has been offered and brought to scale during an acute emergency, showing both the demand for and feasibility of such a response.

Despite the evidence suggesting the need for safe abortion services and consensus in the humanitarian community about the importance of providing comprehensive abortion care, only three peer-reviewed papers published in the past 10 years documenting detailed individual-level abortion experiences of those living in refugee camps or settlements were identified; data from all three papers are drawn from the same research study. In this study on women from the Democratic Republic of Congo who experienced sexual-violence related pregnancies, barriers to termination among those who carried these pregnancies to term included: fear of death from unsafe abortion procedures, lack of knowledge of where to access services, or a failed abortion or ongoing pregnancy after attempting to terminate with herbal remedies (17). Among those who did terminate their pregnancies, the majority used medications (most commonly, quinine) or traditional herbs (most commonly, cimpokolo, or *Phytolacca dodecandra*) obtained on their own or through family, friends, or traditional healers (18). Many reported seeking medical care as a result of their symptoms; it is unknown whether the methods they used were successful on their own, or if participants obtained surgical procedures or other post-abortion care treatment in order to terminate their pregnancies after inducing bleeding. Findings from these studies corroborate other qualitative findings that have found a lack of access to information on abortion in humanitarian settings, and highlight the need for interventions that increase access to information on self-managed safe abortion.

## Potential of Abortion Self-Care in Humanitarian Contexts

Within the humanitarian field, many of the calls for action have focused on overcoming barriers to facility-based abortion care provision (19). While these efforts are critical, this view often centers clinic-based care as the gold standard of abortion provision and ignores the reality that for many, “safety” of abortion care involves more than the location or provider involved (20). Evidence from other settings where SMA is common indicates that fear of mistreatment and stigma from providers, as well as concerns around privacy, are primary drivers for why people choose to self-manage despite the availability of abortion services within the formal healthcare system (21–23). Such concerns are likely heightened during displacement, where known caregivers and community intermediaries are replaced by systems managed by new state actors or international non-governmental organizations.

As a result of misconceptions about the legality of abortion provision, as well as perceived loss of funding or donor unwillingness to support abortion provision, humanitarian organizations responsible for provision of health care services in refugee camps, settlements, or conflict-affected areas, either do not provide abortion services, or are unable to meet the full need for abortion services in these contexts (4, 24). Logie et al. have highlighted the potential of self-care interventions in advancing sexual and reproductive health in humanitarian settings, as they can increase lay health worker capacity and potentially better serve the needs of individuals who face additional marginalization such as adolescents, lesbian, gay, transgender, and gender expansive people, and people with disabilities (25). A growing body of evidence suggests that individuals can safely and effectively manage their abortions if they have access to WHO-approved medications for abortion (misoprostol alone, or misoprostol in combination with mifepristone) and information is available about how to take the pills, confirm abortion completion, and how to recognize warning signs that might warrant follow-up medical care (23, 26). Indeed, the WHO has highlighted the potential of self-care interventions, including SMA, as a strategy that gives individuals greater control over their experience and privacy, while also overcoming challenges such as healthcare worker shortages and high out-of-pocket-costs (6).

Global evidence has demonstrated the safety and effectiveness of a range of models for providing information and support for SMA. *Harm reduction* programs are based within the formal healthcare system, where medical providers provide individuals with information before and after SMA, but do not directly provide individuals with the medications (27). Individuals might access medication from *pharmacies* or informal drug sellers; though the quality of the information that they receive can be variable (28). *Abortion accompaniment networks*, along with *safe abortion hotlines*, are run by lay counselors and feminist activists, and offer individualized evidence-based counseling and support, including information on how to self-assess eligibility for medications, how to procure medications, how to take the medications, how to manage abortion symptoms and assess completion, when to seek healthcare, and offer virtual or in-person guidance and support throughout the process (29, 30). In *community-distribution programs*, community health workers, midwives, or lay providers are trained in providing counseling and support around medication abortion and directly distribute the medications to individuals to ensure quality of the drugs provided. While work by Foster et al. on the Thai-Burma border (31) among Burmese migrants and refugees provides important evidence for the safety, effectiveness, feasibility, and acceptability of this model of abortion care, it is one of the only research studies evaluating any abortion access intervention in a humanitarian context. Additional research is urgently needed to develop appropriate, context-specific interventions that provide information and support to people who are self-managing their abortion through a variety of different models of support (32, 33).

There are many advantages to SMA—such as privacy, confidentiality, and affordability—that contribute to its potential to revolutionize safe abortion access in humanitarian settings. SMA interventions can be tailored to improve access for specific populations who are often not centered in intervention design or service provision, such as young people, LGBTQI individuals, and people with disabilities. Additionally, SMA can reduce the reliance on overburdened health systems, which may further increase access by providing people with an additional option for abortion care. The de-medicalization of this care is likely to be appealing to those who may have been persecuted or discriminated against prior to displacement and are still building trust in their new environments. Although the stigma of abortion is felt in both legally liberal and restrictive settings (34), additional cultural barriers and a loss of power and autonomy experienced by displaced people certainly magnifies these concerns.

However, there are additional considerations that are specific to humanitarian contexts. Which abortion medications are available and how are they accessed? How does access to water and sanitation facilities—especially shared toilets, lack of clean water, sanitary pads or cloths, which may make managing the products of conception and bleeding onerous and difficult to hide—affect abortion experiences? What are the impacts of poverty and a lack of access to cash, which can make purchasing abortion medications, pain control medications and hygiene materials, difficult decisions when placed against other individual and household needs? How does crowded housing and lack of privacy from other members of the household influence individual decisions around abortion methods and care seeking? What are the legal contexts, how are they understood and what are the contextual effects? These and other issues highlight the critical importance of empirical research on direct abortion experiences to understand the needs, barriers, and facilitating factors around abortion self-care.

## Discussion

Despite the many calls for additional research, funding, and attention toward provision of safe abortion services for those living in humanitarian contexts, little is known about the abortion experiences of individuals in these contexts. No peer-reviewed evidence exists on the incidence of abortion in any humanitarian context, nor have any studies sought to rigorously assess the information needs, knowledge gaps, or experiences with abortion among those living in protracted humanitarian emergencies, an increasingly common situation as most displaced people now spend over 17 years of their lives in displacement. Limited evidence has suggested that women in humanitarian settings often resort to using unsafe methods to terminate their pregnancies, and that a substantial proportion of maternal mortality in such settings may be related to complications from unsafe abortion. Even in contexts where health-implementing organizations are providing comprehensive abortion services, lack of knowledge, fear of legal repercussions, and abortion stigma may prevent people from accessing care from these providers.

Given the potential of SMA to revolutionize access to abortion in a variety of settings, including in humanitarian settings, additional research exploring the barriers and facilitators for SMA is sorely needed. For example, inclusive research should seek to understand what information people need, how it should be delivered, what their preferences are around support during their process, how to support linkages to formal healthcare systems when needed or desired, how to center concerns about privacy and individual legal considerations depending on the context, and how and where people are sourcing the medications and the medication quality, among others.

Efforts to increase information and support for SMA should occur in tandem with efforts to strengthen facility-based abortion care. While SMA interventions can reach multitudes of people with lifesaving information long before humanitarian agencies have the political will and technical abilities to provide this care, humanitarian agencies and advocates should renew and strengthen their efforts to make facility-based abortion care accessible, as individuals not only deserve the right to have an abortion, but to decide where, how, and with what support their abortion takes place.

There is a human rights imperative to expanding and ensuring global abortion access—and those living in humanitarian contexts should not be overlooked. Interventions that support people who are self-managing their abortion have the potential to increase both the extent and the quality of abortion access in these settings; future research efforts should focus on centering the information needs and priorities of individuals in need of safe abortion care in these contexts to inform the development of person-centered interventions.

## Data Availability Statement

The original contributions presented in the study are included in the article/supplementary material, further inquiries can be directed to the corresponding author/s.

## Author Contributions

RJ, BP, TF, CG, RO, YW, EW, and UR conceptualized the perspective presented in this manuscript. RJ, BP, and TF drafted the manuscript. CG, EW, JK, RO, YW, and UR provided critical reviews and additions to the manuscript. All authors contributed to the article and approved the submitted version.

## Conflict of Interest

The authors declare that the research was conducted in the absence of any commercial or financial relationships that could be construed as a potential conflict of interest.

## Publisher's Note

All claims expressed in this article are solely those of the authors and do not necessarily represent those of their affiliated organizations, or those of the publisher, the editors and the reviewers. Any product that may be evaluated in this article, or claim that may be made by its manufacturer, is not guaranteed or endorsed by the publisher.
